# Using dimensionless numbers to understand interfacial mass transfer for parallel flow in a microchannel

**DOI:** 10.1007/s10404-025-02828-1

**Published:** 2025-07-09

**Authors:** Anand Sudha, Martin Rohde

**Affiliations:** Radiation Science and Technology, Technische Universiteit Delft, Mekelweg 5, 2628 CD Delft, The Netherlands

**Keywords:** Transport phenomena, Mass transfer, Kinetics, Parallel flow

## Abstract

Liquid-liquid Extraction has emerged as a major technique for radioisotope extraction in recent years. This technique is particularly advantageous in the microscale as the surface-volume ratio is much larger. Since some of these radioisotopes have short half-lives, parallel flow in the microscale is used to extract them as it eliminates the need for separating the two fluids. Though such a configuration has been experimentally studied, dimensionless numbers have not been employed to understand the mass transfer mechanisms. This study uses three dimensionless numbers—the Biot, Peclet and Damkohler numbers—to delve deeper into mass transfer with a chemical reaction at the interface. Mass transfer simulations are performed using a Finite Difference model to solve the 2D Convection-Diffusion Equation with a first-order reaction at the interface, and these numbers are varied. The Damkohler number was observed to have the maximal impact on the extraction efficiency, and this was confirmed to be the case when the extraction efficiency didn’t change much as long as the Damkohler number was kept constant. In general, a higher Damkohler number results in a higher extraction efficiency and a correlation was proposed to quantify this influence.

## Introduction

Radioisotopes have become increasingly important in the past few decades because of their vast capabilities in medical applications (Czernin et al. 2019), particularly in the diagnosis (Veall et al. 1958; Shapiro et al. 2001), imaging (Huang et al. 2015; Pedersen et al. 2018) and treatment of cancer and other diseases (Sgouros et al. 2020; Vandergrift and Patel 2006). For these applications, the radioisotopes have to be highly pure in accordance with the medical standards (Shivarudrappa and Vimalnath 2005). However, they are normally present in a mixture of oxides along with fission products generated from the nuclear reaction (Mariet et al. 2019). Pure radioisotopes are not easy to obtain because of the presence of various chemical products in the nuclear fuel.

In order to extract the maximum utility from a radioisotope, it is necessary to separate it from the fuel to render it safe for medical applications. Thus, the desired radioisotope needs to be extracted from the product at high purity as impurities reduce the efficacy of the drug or diagnosing technique (Martini et al. 2019a).

Liquid-liquid Extraction (LLE) is an important technique for radioisotope extraction and purification Martini et al. (2019b); Assmann and Ładosz (2013). This is traditionally carried out on a batch scale, where the multiphase mixture is agitated in large-scale containers or columns followed by gravity-based separation of the two phases. However, this is not very favourable for isotope extraction because large volumes of fluids are required, thus leading to a low surface-volume ratio, which in turn increases the time required for successful mass transfer (Su et al. 2010; Martini et al. 2019a). Additionally, a separation step is necessary to obtain pure radioisotopes, and this can be extremely detrimental when transferring radioisotopes with short half-lives since the time required for an extra step might lead to a significant decay in isotope concentration (Martini et al. 2019a). If the concentration of the solute is low, batch extraction is very difficult as small concentrations of solutes have to cover large distances to diffuse through the interface (Nichols et al. 2011).

These limitations can be overcome by shifting the process to the microscale. In addition to using far smaller liquid volumes, this form of extraction largely increases the surface-to-volume ratio (Su et al. 2010), with some microfluidic devices showing an almost tenfold increase (Ghaini et al. 2010; Holbach and Kockmann 2013). This also has the effect of decreasing the diffusion distances the isotope has to traverse while also stabilizing the liquid-liquid interface. The microchannel can be modified geometrically to enhance mass transfer and is amenable to automation, thus allowing greater control over the transport phenomena (Hibara et al. 2001; Tokeshi et al. 2002).

Two flow patterns in microchannels have been used for extraction studies—slug and parallel flow. The surface-to-volume ratio is the largest in slug flow, which makes it particularly suited for mass transfer. Furthermore, the circulation that occurs inside the slugs in the form of Taylor vortices caused by friction on the wall increases the extraction efficiency (Kashid et al. 2005). This is why many studies have been devoted to slug flow (Zhang et al. 2015; Soh et al. 2017; Raimondi and Prat 2011). However, slug flow is not advantageous when extracting radioisotopes because it necessitates a separation step which usually involves membranes (Kralj et al. 2007) or complex geometries to selectively trap droplets of one phase (Shen et al. 2006). If the radioisotopes have short half-lives, the separation step could significantly limit the viable amount of radioisotope (Goyal et al. 2014).

This is the main reason why parallel flow with the interface located exactly at the centre of the channel is very important for radioisotope extractions and is used in different studies (Goyal et al. 2014; Foroozan Jahromi et al. 2018; Farahani et al. 2021). Considering the utility of parallel flow in radioisotope extractions, the mass transfer mechanisms for such a regime also need to be understood if microfluidic LLE is to be applied in different applications.

In the case of multiphase flow, mass transfer can be influenced solely by diffusion, a chemical reaction, or a combination of both (Malengier et al. 2012; Ciceri et al. 2014; Karim et al. 2023). Generally, binding agents or chelators are added to the extracting fluid to enhance mass transfer through a chemical reaction (Hellé et al. 2014; Ciceri et al. 2013; Haroun et al. 2010). These chemical reactions can either take place across the whole extracting fluid (Zhang et al. 2019; Haroun et al. 2010) or be restricted only to the region around the liquid-liquid interface (Hellé et al. 2014; Ciceri et al. 2014). The former is normally restricted to slug flow as the flow circulation near the slug boundaries transports the solute and ions across the entire slug (Zhang et al. 2019).

However, for parallel flow, such circulating flows are generally not observed near the interface. Therefore, the reaction usually occurs in the interface region after which it gradually diffuses to the rest of the extracting fluid (Hellé et al. 2014; Karim et al. 2023). Considering the prevalence of such a reaction mechanism in microfluidic LLE (Karim et al. 2023; Ciceri et al. 2014, 2013), it is important to understand the forces and factors that influence such a mass transfer.

Multiple papers have studied LLE for parallel flow using models (Vir et al. 2014; Žnidaršič-Plazl and Plazl 2007; Ramji and Pushpavanam 2019) to solve the Convection-Diffusion Equation (CDE) with species interface transfer, but these studies only considered the influence of diffusion and convection. Many experimental studies involving radioisotopes utilize chemical reactions to speed up the process of radioisotope transfer (Ciceri et al. 2014; Jovanović et al. 2012; Goyal et al. 2014; Hellé et al. 2014). Thus, it is important to understand the competing phenomena driving this kind of mass transfer. Dimensionless numbers are very useful in providing an understanding of the transport phenomena as they show the competing forces involved. Zhang et al. (2019) numerically studied the extraction mechanism of Lanthanum for slug flow using the Damkohler (Da) and Graetz (Gz)numbers. In this way, they could better understand how the reaction takes place inside the slug and how the flow velocity subsequently influences the reaction, thereby the mass transfer. To the authors’ knowledge, similar studies for reactions taking place close to the interface have not included dimensionless numbers in their analysis for parallel flow. This limits the understanding and applicability of the results as they are restricted to a particular set of fluids and solutes, and do not provide insight into the forces that influence the mass transfer mechanisms. Karim et al. (2023) mentioned the important dimensionless numbers for interfacial mass transfer, but did not use them in their study.

Therefore, this study seeks to understand the factors that influence interfacial mass transfer. For this purpose, numerical simulations are conducted for the set of fluids and isotopes used by Hellé et al. (2015). Once the model is validated, dimensionless numbers are varied to understand the effect of the competing forces on the mass transfer for the same fluids used by Hellé et al. (2015).

This paper is organized as follows. First, the theory behind the model and mass transfer equations is discussed in Sect. 2. Next, the model is validated by comparing it with the results of Hellé et al. (2015), and then the influence of dimensionless numbers is studied. Finally, the paper is concluded in Sect. 5.

## Theory

**Fig. 1:**
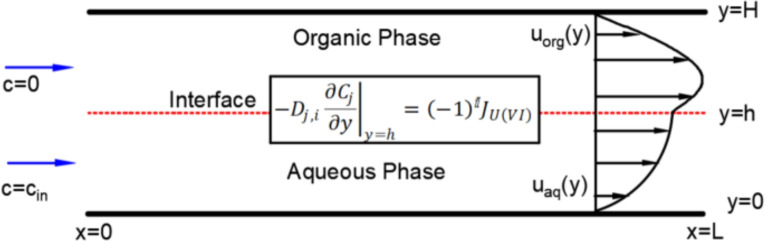
Mass transfer for parallel flow along the *x* direction. The interface is located at $y=h$ and the velocity profiles for each phase are also shown. The species is located in the aqueous phase and will be transferred to the organic phase. A chemical reaction occurs at the interface and an interfacial boundary condition is applied accordingly. *D* corresponds to the diffusion coefficient, C the concentration and *J* the reactive flux at the interface

The transport of species in multiphase flow is governed by the Convection-Diffusion Equation (CDE) Haroun et al. (2010); Kuzmin (2010). The conservative form of the CDE describing the concentration of species *i* is given by:

1 $$\begin{aligned} \underbrace{\frac{\partial c_i}{\partial t}}_{\text {Transient term}} = \nabla \cdot (\underbrace{D\nabla c_i}_{\text {Diffusive}} -\underbrace{\textbf{u}c_i}_{\text {Convective}})+ \underbrace{S_i}_{\text {Source/Sink}} \end{aligned}$$

where $c_i$ is the concentration of the species *i*, *D* is the diffusion coefficient, $\textbf{u}$ is the velocity of the fluid and $S_i$ is the source/sink term which corresponds to the generation/destruction of the species. Both the convective and diffusive terms compete with one another, and a measure of the dominant phenomenon is given by the Peclet number, which is the ratio of convective and diffusive mass transfer (Malengier et al. 2011).

2 $$\begin{aligned} Pe = \frac{uL}{D} \end{aligned}$$

where *L* is the characteristic length of the flow and *u* is the characteristic velocity of the fluid. Equation 1 is solved based on the approximations and boundary conditions prescribed for a given problem. In this work, parallel flow is considered. Figure 1 illustrates the mass transfer case for 2D parallel flow. The species is located in the aqueous phase and will be extracted to the organic phase. To solve Eq. 1 for parallel flow, the following assumptions and approximations are made:

1. Flow is laminar, steady and fully developed.
2. The case is studied in two dimensions.
3. The diffusion along the *x* direction can be neglected. This is because the *Pe* is of $\mathcal {O}$(10$^4$), which indicates that convective transport dominates over diffusive transport.
4. Convection along the *y* direction can be neglected, as the velocity along the lateral directions is negligible in the case of parallel flow.
5. The interface is assumed to be straight even though it is curved in reality by a few microns.

Based on the figure and these assumptions, Eq. 1 can be simplified to the following form:

3 $$\begin{aligned} \textbf{u} \frac{\partial c}{\partial x}= D \frac{\partial ^2 c}{\partial y^2} \end{aligned}$$

Since the reaction takes place primarily at the interface and does not occur across the rest of the channel (will be discussed in more detail in Sect. 2.1), the source term in Eq. 1 can be neglected. The boundary conditions (BC) applied are:

1. No species penetration at the adiabatic walls
2. Given concentration profile at the inlet

The no-penetration BC ensures that there is no diffusion across the wall and is given by:

4 $$\begin{aligned} \begin{aligned} \frac{\partial c}{\partial y}=0, \ \ \textrm{at} \ y=0 \ \text {and} \ y=H\\ \end{aligned} \end{aligned}$$

At the inlet, the following boundary condition is applied:

5 $$\begin{aligned} \begin{aligned} c=c_{\textrm{in}}, \ \ \ \ x=0, \ 0 \le y \le h \\ c=0, \ \ \ \ x=0, \ h \le y \le H \\ \end{aligned} \end{aligned}$$

For the problem to be fully bounded, a boundary condition needs to be defined at the interface. When the chemical reaction takes place only at the interface and does not extend to the individual phases as observed in the experiments of Hellé et al. (2015), a boundary condition incorporating the chemical reaction must be applied at the interface. Hellé et al. (2015); Ciceri et al. (2014).

### Interfacial boundary condition

In this paper, numerical simulations are conducted using the fluids and kinetics of Hellé et al. (2015). Their extraction kinetics involved the transfer of Uranium from the aqueous to the organic phase. The chemical reaction describing the formation of Uranium is given by:

$$\text {UO}_{2}\text {(Cl)}{_{4}{^{2-}}} + 2\,\text {R}_{4}\text {N}^{+},\text {Cl}^{-} \rightleftarrows (\text {R}_{4}\text {N}^{+})_2\text {UO}_{2}({\text {Cl})_{4}{^{2}}}^{-} + 2\text {Cl}^{-}$$

Uranium is extracted from the aqueous HCl phase to the organic (dodecane) phase using Aliquat ($2\,\text {R}_{4}\text {N}^{+},\text {Cl}^{-}$) to form a new Uranium complex that can only dissolve in the organic phase. R corresponds to an alkyl group here. It is assumed that the aqueous chloro-uranium complex does not diffuse into the organic phase and the Aliquat does not diffuse to the aqueous phase. The concentrations of Aliquat and $\hbox {Cl}^{-}$ are also much larger than the Uranium concentrations, therefore they can be considered constant during the reaction. Since the Aliquat and $\hbox {Cl}^{-}$ do not diffuse into the aqueous and organic phase, respectively, we can eliminate the source term from Eq. 1 as the reaction takes place mainly at the interface. This is in contrast to studies involving a source term, where the reaction takes place across the entire phase, as in the case of slug flow (Zhang et al. 2019). Experiments involving radioisotope transfer have also confirmed this to be the case for parallel flow (Ciceri et al. 2014; Hellé et al. 2015; Trapp et al. 2024). The interfacial BC for the Uranium species is given by Ciceri et al. (2014); Hellé et al. (2015):

6 $$\begin{aligned} -D_{j,i}\left. \frac{\partial c_j}{\partial y}\right| _{y=h}= (-1)^i J_{\mathrm {U(VI)}} \end{aligned}$$

where *j* corresponds to the species, which is just Uranium (VI) in this case, *i* is the phase, with $i=1$ being the aqueous phase and $i=2$ being the organic phase, $D_{j,i}$ is the diffusion coefficient of species *j* in phase *i*, *h* is the position of the interface as shown in Fig. 1 and $J_{\mathrm {U(VI)}}$ is the local mass flux (mol/($\hbox {m}^2$s)) of Uranium (VI) generated at the interface because of the chemical reaction. For this first-order reaction with the above assumptions, the local flux at the fluid-fluid interface is given by:

7 $$\begin{aligned} J_{\mathrm {U(VI)}}= K_1 \left[ \text {UO}_{2}(\text {Cl})_4{^{2-}} \right] -K_{-1}\left[ 2\, \text {R}_{4}\text {N}^{+},\text {Cl}^{-}\right] \end{aligned}$$

where $K_1$ and $K_{-1}$ (m/s) are the forward and reverse reaction kinetic constants respectively. The above equation can be rewritten as:

8 $$\begin{aligned} J_{\mathrm {U(VI)}}= K_1 \left( \left[ \text {UO}_{2}(\text {Cl})_4{^{2-}} \right] -\frac{1}{K_{\textrm{eq}}}\left[ 2\, \text {R}_{4}\text {N}^{+},\text {Cl}^{-}\right] \right) \end{aligned}$$

where ${K_{\textrm{eq}}}$ is the equilibrium kinetic constant, which is nothing but the ratio of the forward and reverse kinetic constants (Ciceri et al. 2014).

9 $$\begin{aligned} {K_{\textrm{eq}}}= \frac{K_1}{K_{-1}} \end{aligned}$$

### Numerical modeling

The CDE in this paper is solved using a 2D Finite Difference Method (FDM). FDM is a numerical technique that solves differential or partial differential equations by approximating derivatives using finite differences (Ames 2014). These finite differences are usually evaluated using Taylor’s series expansion at the point of consideration. The physical space is first discretized into nodes in a grid. For solving Eq. 3, the backward FD is applied along the x direction and central FD is applied for all the other derivatives, similar to the method used by Malengier et al. (2011). The central FD is applied in the y direction because of its 2nd order convergence. Since the inlet condition is applied at $x=0$, it was more convenient to apply backward FD along the x direction instead of defining a ghost node for central FD. If we consider the 2D case, Eq. 3 is discretized as follows:

10 $$\begin{aligned} u_{i+1,j} \frac{c_{i+1,j}-c_{i,j}}{\Delta x}= D \frac{c_{i+1,j+1} - 2c_{i+1,j} + c_{i+1,j-1}}{\Delta y^2} \end{aligned}$$

Here, *i* corresponds to the nodes along the x direction and *j* corresponds to the nodes along the y direction. A grid length of 2.5 $\mu$m was used for both directions and grid convergence was obtained at the same length.

### Dimensionless numbers

For this case of diffusion with a reaction at the interface, two dimensionless numbers can be used to describe the transport phenomena (Karim et al. 2023). One is the Peclet number, which describes the ratio of the convective transport to the diffusive transport rate (Seader 2006). The formula for this number along the axial or *x* direction was given in Eq. 2. For a microfluidic channel, the Peclet number along the transverse direction in terms of the flow rate is given by:

11 $$\begin{aligned} Pe= \frac{Q}{DL} \end{aligned}$$

where *Q* is the flow rate of the aqueous/organic phase, *L* is the length of the channel, and *D* is the diffusion coefficient. This equation can be further simplified for the 2D case (Karim et al. 2023):

12 $$\begin{aligned} Pe_{\textrm{2D}}= \frac{uH^2}{DL} \end{aligned}$$

where *u* is the characteristic velocity of the fluid and *H* is the width of the channel. The characteristic velocity in this case is nothing but the inlet velocity of each fluid, with the characteristic length scales being the channel length *L* for convection and channel width *H* for diffusion. To quantify the competing influences of the interfacial reaction and diffusion, the Biot number is used (Karim et al. 2023). The Biot number is chosen over other commonly used dimensionless numbers such as the Sherwood number to describe the influence of the reaction kinetics in comparison with diffusion. The Sherwood number looks at the ratio of total mass transfer rate to the diffusion (Gervais and Jensen 2006; Farahani et al. 2021), and this is less useful in this study as it doesn’t describe the individual impacts of reaction or convection in relation to diffusion. Since we wish to quantify each of these forces individually, the Biot number is a better option as it clearly characterizes the competing influences of reaction and diffusion. This is especially true if we consider our case of interfacial mass transfer, where the kinetics of the reaction is mainly governed by the phenomena taking place at the interface (Karim et al. 2023). The Biot number is given by:

13 $$\begin{aligned} Bi=\frac{K_1 H}{D} \end{aligned}$$

The equilibrium kinetic constant (Eq. 9) is also an important dimensionless number for mass transfer by reaction as it indicates the propensity of the reactants to form the products. In addition to these three dimensionless numbers, we employ a dimensionless number that looks at the influences of the interfacial reaction and convection for this mass transfer. Though this number exists, it hasn’t been applied in this context before as far as the authors’ knowledge is concerned. The Damkohler number is defined as the ratio of residence time to the reaction time scale (Fogler 2010). For our case, the residence time is the convection time scale, so the convective Damkohler number ($Da_{\textrm{c}}$) is the ratio of the Biot and Peclet numbers.

14 $$\begin{aligned} Da_{\textrm{c}}= \frac{Bi}{Pe}= \frac{K_1HL}{Q} \end{aligned}$$

In the case of 2D, this equation simplifies to:

15 $$\begin{aligned} Da_{\textrm{c}}=\frac{K_1L}{uH} \end{aligned}$$

### Flow modelling

Based on the assumptions discussed earlier, the simplified Navier–Stokes equations can be solved for two-phase parallel flow in a rectangular channel (Hellé et al. 2015; Ciceri et al. 2014; Malengier et al. 2011):

16 $$\begin{aligned} \frac{dp}{dx}= \mu \frac{d^2u}{dy^2} \end{aligned}$$

Instead of solving this equation using simulations, the velocity profile is obtained using the analytical Poiseuille solution for the velocity (Rapp 2022; Malengier et al. 2012) in 2D The main reason for this is that the contact angle used by Hellé et al. (2015) is unknown, so it is difficult to obtain the exact position of the interface from the simulations. An advantage of using the analytical solution is that we can fix the position of the interface to lie exactly at the centre of the channel, instead of adjusting the contact angle in simulations to determine the appropriate contact angle that matches Helle et al’s experiments. Though studies such as those by Vir et al. (2014) and Ramji and Pushpavanam (2019) have studied the influence of interface position on extraction efficiency, it is not practical in the case of radioisotope transfer. This is because we wish to ensure stable parallel flow without any leakage to the other outlets (Trapp et al. 2024; Ban et al. 2011; Aota et al. 2009; Sudha et al. 2025). If the interface position is not at the centre, a separation step would be needed to ensure maximal purity. Considering the low half-lives of the radioisotopes used, this would limit the amount of radioisotope available for application. Thus, this study uses the analytical Poiseuille flow solution to fix the interface position at the centre. A 2D geometry is also selected for this purpose, as the 2D analytical solution can ensure that the interfacial position is fixed. The analytical 3D velocity flow profile is determined using partial Eigen expansions, which do not include the position of the interface. These Eigen constants have to be determined using our enforced boundary conditions, which involve a bit of trial-and-error (Malengier et al. 2012; Vir et al. 2014). To avoid the problems associated with the selection of the Eigen constants, we choose to implement our results in 2D. A slight error in the choice of constants results in errors in the velocities at the boundaries. Additionally, Vir et al. (2014) showed that the 2D results are similar to the 3D results for channels with higher aspect ratios, which is the case for our channel. The velocity profiles of the two fluids in 2D are given by Malengier et al. (2011):

17 $$\begin{aligned} {\left\{ \begin{array}{ll} u_{\textrm{aq}} (y) = - \frac{y(h^2(\mu _{\textrm{org}}-\mu _{\textrm{aq}})-yH\mu _{\textrm{aq}}+H^2\mu _{\textrm{aq}}-yh(\mu _{\textrm{org}}-\mu _{\textrm{aq}}))}{2(h(\mu _{\textrm{org}}-\mu _{\textrm{aq}})+H\mu _{\textrm{aq}})\mu _{\textrm{aq}}} \nabla P \ \ \textrm{for} \ 0< y< h, \\ u_{\textrm{or}}(y) = -\frac{(H-y)(Hh(\mu _{\textrm{org}}-\mu _{\textrm{aq}})+yH\mu _{\textrm{aq}}-h^2(\mu _{\textrm{org}}-\mu _{\textrm{aq}})+yh(\mu _{\textrm{org}}-\mu _{\textrm{aq}}))}{2(h(\mu _{\textrm{org}}-\mu _{\textrm{aq}})+H\mu _{\textrm{aq}})\mu _{\textrm{aq}}} \nabla P \ \ \textrm{for} \ h<y<H \end{array}\right. } \end{aligned}$$

where *u*(*y*) is the velocity profile of the fluid with the subscripts aq and org corresponding to aqueous and organic phases respectively, *h* is the position of the interface, *H* is the width of the channel, $\mu$ is the viscosity, and $\nabla P$ is the imposed pressure gradient which remains constant across the channel. The influence of surface tension and contact angle is absent from these equations, and as both these quantities have a role to play in the position of the interface, the analytical flow profile is an approximation. Despite this, it can be useful in studying the effect of the dimensionless numbers on the transport phenomena as the interface position can be fixed. If the flow rate of one fluid per unit depth and the position of the interface are fixed, the pressure gradient and the flow rate of the other fluid per unit depth can be determined using the following formulae (Malengier et al. 2011):

18 $$\begin{aligned} {\left\{ \begin{array}{ll} Q_{\textrm{aq}}= \int _0^h u_{\textrm{aq}} (y) dy \ = - \frac{h^2(h^2 \mu _{\textrm{org}}-h^2\mu _{\textrm{aq}}-2Hh\mu _{\textrm{aq}}+3H^2\mu _{\textrm{aq}})}{12(\mu _{\textrm{org}}h-\mu _{\textrm{aq}}h+\mu _{\textrm{aq}}H)\mu _{\textrm{aq}}} \nabla P \\ Q_{\textrm{org}}= \int _h^H u_{\textrm{org}}(y) dy \\ = - \frac{(4\mu _{\textrm{org}}hH^3-\mu _{\textrm{org}}h^4-4\mu _{\textrm{aq}}hH^3+\mu _{\textrm{aq}}h^4+\mu _{\textrm{aq}}H^4-4\mu _{\textrm{aq}}Hh^3-9\mu _{\textrm{org}}h^2H^2+6\mu _{\textrm{aq}}h^2H^2+6\mu _{\textrm{org}}Hh^3)}{12\mu _{\textrm{org}}(\mu _{\textrm{org}}h-\mu _{\textrm{aq}}h+\mu _{\textrm{aq}}H)} \nabla P \end{array}\right. } \end{aligned}$$

where *Q* is the 2D flow rate.

## Fluid properties for simulations

The simulations use the same fluids of Hellé et al. (2015), where Uranium isotope was extracted from an aqueous HCl solution to n-dodecane solution. The properties of the fluids along with the diffusion coefficient of Uranium are described in Table 1.

**Table 1:** Flow, diffusion and kinetic properties used in the simulations

**Property**	**Aqueous Phase**	**Organic Phase**
Density (kg/$\hbox {m}^3$)	1081	750
Viscosity (mPas)	1.27	1.48
Surface Tension (mN/m)	29.83	–
Diffusion Coefficient ($\hbox {m}^2$/s)	10$^{-8}$	10$^{-9}$
$K_1$ (m/s)	1.6 $\times 10^{-5}$	–
$K_{\textrm{eq}}$	–	5.91

## Results

### Validation

The mass transfer simulations are performed in the rectangular channel shown in Fig. 1, with the dimensions of the channel being 8 cm $\times$ 100 $\mu$m $\times$ 40 $\mu$m. First, the model needs to be validated by comparing it with the results of Hellé et al. (2014). We use only the 2D geometry in our simulations, thereby only taking the length and width of the rectangular channel. Hellé et al. (2015) conducted their simulations in 3D. The Extraction Efficiency (EE) is determined here and is used to characterize the efficiency of mass transfer. It is calculated as follows:

19 $$\begin{aligned} \textrm{EE}= \frac{C_{\textrm{org,o}}}{(C_{\textrm{org,o}}+C_{\textrm{aq,o}})} \times 100 \end{aligned}$$

where *C* is the concentration of Uranium isotope, the subscripts org and aq correspond to the organic and aqueous phases respectively, with o corresponding to the outlet. Our simulations are compared with their experiments for three different channel lengths using the analytical Poiseuille solution described in Sect. 2.4. The results are described in Table 2.

**Table 2:** Comparison of numerical simulations with the experiments of Hellé et al. (2015). The subscript sim corresponds to simulations

Channel Length (cm)	$\mathbf {Q_{\textrm{aq}}}$ ($\mu$L/min)	$\mathbf {Q_{\textrm{org}}}$ ($\mu$L/min)	$\mathbf {Q_{\textrm{aq,sim}}}$ ($\mu$L/min)	$\mathbf {Q_{\textrm{org}}}$ ($\mu$L/min)	**EE**(%)	$\hbox {EE}_{\textrm{sim}}$(%)
8	1.56	1.38	1.56	1.5	76.3	76.91
12	1.56	1.38	1.56	1.5	85.3	84.72
20	1.56	1.38	1.56	1.5	86.12	85.34

In the simulations, the aqueous flow rate and the interface position are fixed at $Q_{\textrm{aq, sim}}$ = 1.56 $\mu$L/min and $h= H/2$ respectively. This corresponds to the results observed by Hellé et al. (2015). However, using the analytical solution leads to a slight change in the organic flow rate as seen in Table 2. This doesn’t result in a significant change in the extraction efficiency though. Since our study looks to understand the influence of dimensionless numbers on interfacial mass transfer, the analytical profile is very useful as we can easily change the velocity magnitude and observe its impact on the mass transfer. Therefore, for the purposes of this study, the code is sufficiently validated.

**Fig. 2:**
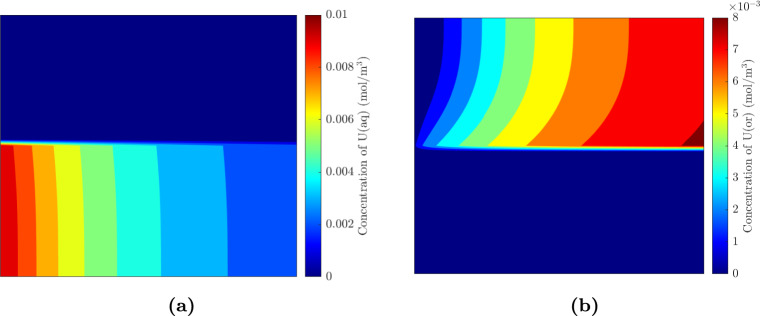
Simulated concentration profiles using the data from Hellé et al. (2015) and the analytical flow profile defined in Eq. 17. $Q_{\textrm{aq}}$= 1.56 $\mu$L/min, $h=H/2$, $Bi_{\textrm{aq}}$=0.16, $Bi_{\textrm{or}}$=1.6, $Pe_{\textrm{aq}}$=0.081, $Pe_{\textrm{or}}=0.78$. **a** Aqueous concentration profile. **b** Organic concentration profile

The concentration profiles obtained from our simulations for a channel length of 8 cm are shown in Figs. 2a and 2b. The obtained concentration profile is similar to that observed by Hellé et al. (2015) in their experiments. The concentration of Uranium in the aqueous phase is maximum at the inlet, and as we move along the length of the channel, Uranium is more efficiently transferred to the organic phase. The maximum Uranium concentration in the organic phase is naturally observed to be at the end of the channel, which is the case with Hellé et al. (2015) experiments as well. At the end of the channel, we can see that the concentration of Uranium is larger closer to the interface. This is because the reaction takes place at the interface, and the remaining Uranium has to diffuse along the width of the channel. The influence of dimensionless numbers is discussed in the following sections.

### Effect of Bi, Pe and $K_{\textrm{eq}}$

The influence of the Biot and Peclet numbers on extraction efficiency is studied in this subsection. The microfluidic channel used by Hellé et al. (2015) is used here, with the parameters the same as that described in Table 1 unless otherwise stated. Hellé et al. (2015) only considered the accuracy of their model in their paper and did not look at the influence of diffusion coefficients, so we use their channel dimensions and chemical properties to understand the role of the competing forces involved in the transport phenomena. We vary the Bi by varying $K_1$ (Eq. 13) as changing *D* or *H* also influences the Peclet number (Eq. 12). $K_{\textrm{eq}}$ remains the same for all Bi ($K_{\textrm{eq}}$=5.9). The flow rate of the aqueous phase is the same as the one used in Fig. 2 ($Q_{\textrm{aq}}$ = 1.56 $\mu$L/min), with the position of the interface fixed at the middle of the channel.

**Fig. 3:**
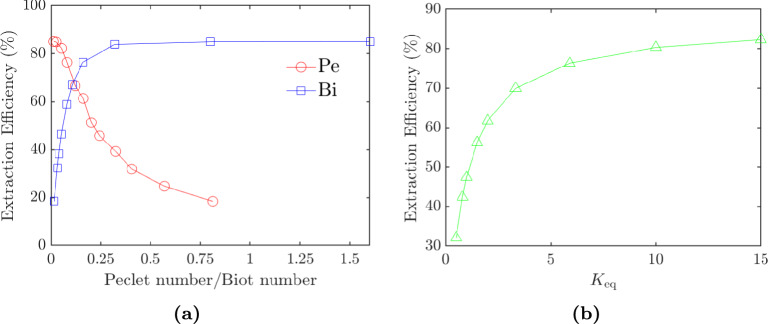
Influence of **a** Peclet and Biot numbers, and **b** $K_{\textrm{eq}}$ on the extraction efficiency in the microfluidic channel used by Hellé et al. (2015). Bi is varied by changing $K_1$, Pe by changing the flow rate/velocity and $K_{\textrm{eq}}$ is varied by changing $K_2$. The Peclet and Biot number of the aqueous phase is shown here

Similarly, we vary the Pe number by varying either the velocity or length of the channel to ensure that only the Pe, and not the Bi number, is changed. The interface position is fixed at the middle of the channel regardless of the flow rate. The extraction efficiencies are determined and the results are plotted for varying Pe and Bi in Fig. 3a.

As expected, a larger Bi and a smaller Pe number leads to large extraction efficiencies. A fast reaction ensures that the Uranium product forms quickly, and a lower velocity allows for more time for Uranium to diffuse towards the reactive interface (Karim et al. 2023; Gervais and Jensen 2006). At high Pe numbers, Uranium does not have enough time to diffuse to the interface.

From Fig. 3a, we can see that saturation in extraction efficiency is observed after $Bi_{\textrm{aq}}$ =0.32. At such Bi numbers, the system is now diffusion-limited, and a higher extraction efficiency can be obtained only when we operate at smaller Pe numbers. Similarly, at low Pe numbers, saturation is observed as the system is now reaction-limited (Gervais and Jensen 2006).

Finally, the influence of $K_{\textrm{eq}}$ on the extraction efficiency is plotted in Fig. 3b. $K_{\textrm{eq}}$ is varied by only changing $K_{-1}$, thereby ensuring that the Biot number remains the same ($Bi_{\textrm{aq}}$=0.16 and $Pe_{\textrm{aq}}$=0.081). As expected, a higher $K_{\textrm{eq}}$ results in a higher extraction efficiency, and this is because there is a greater propensity for the formation of the Uranium complex in the organic phase.

### Effect of $Da_{\textrm{c}}$

#### Influence of the Diffusion Coefficient

A higher $Da_{\textrm{c}}$ is preferred for efficient mass transfer because this corresponds to a higher Bi or/and lower Pe. From Eq. 14, we can see that $Da_{\textrm{c}}$ increases as we increase $K_1$ and decreases with increase in velocity. These two parameters affect both *Bi* and *Pe* (Eqs. 13 and 12), therefore, to understand the influence of $Da_{\textrm{c}}$, the diffusion coefficient and width of the channel are varied. The diffusion coefficient affects both Pe and Bi, but $Da_{\textrm{c}}$ remains the same. The influence of the diffusion coefficient on the extraction efficiency is tabulated in Table 3, along with the various dimensionless numbers.

**Table 3:** Influence of diffusion coefficient of aqueous and organic on extraction efficiency

$\mathbf {D_{\textrm{aq}}}$ ($\hbox {m}^2$/s)	$\mathbf {D_{\textrm{org}}}$ ($\hbox {m}^2$/s)	$\mathbf {Pe_{\textrm{aq}}}$	$\mathbf {Pe_{\textrm{org}}}$	$\mathbf {Bi_{\textrm{aq}}}$	$\mathbf {Bi_{\textrm{org}}}$	$\mathbf {Da_{\textrm{c,aq}}}$	$\mathbf {Da_{\textrm{c,org}}}$	EE(%)
10$^{-7}$	10$^{-8}$	0.0081	0.078	0.016	0.16	1.98	2.05	77.27
10$^{-7}$	10$^{-9}$	0.0081	0.780	0.016	1.60	1.98	2.05	76.71
10$^{-8}$	10$^{-9}$	0.0810	0.780	0.160	1.60	1.98	2.05	76.31
10$^{-8}$	10$^{-8}$	0.0810	0.078	0.160	0.16	1.98	2.05	76.94
10$^{-8}$	10$^{-10}$	0.0810	7.800	0.160	16.0	1.98	2.05	70.87
10$^{-9}$	10$^{-9}$	0.8100	0.780	1.600	1.60	1.98	2.05	72.95
10$^{-9}$	10$^{-8}$	0.8100	0.078	1.600	0.16	1.98	2.05	73.51
10$^{-9}$	10$^{-10}$	0.8100	7.800	1.600	16.0	1.98	2.05	67.87
10$^{-9}$	10$^{-8}$	0.8100	0.078	1.600	0.16	1.98	2.05	73.52

It appears that the diffusion coefficient has little impact on the extraction efficiency, as the obtained values in Table 3 are very close to each other. In Fig. 3a, we observed that a low Pe or high Bi number results in a higher extraction efficiency. When the diffusion coefficient is high, the Pe number is lower but so is the Bi number. The table covers the regions of low Pe and low Bi number, high Pe and high Bi number, and regions where the Pe and Bi numbers are neither high nor low. This is why $Da_{\textrm{c}}$ is a useful dimensionless number for understanding transport phenomena. From Table 3, it can be seen that $Da_{\textrm{c}}$ remains constant even though Bi and Pe keep changing, and this is because $Da_{\textrm{c}}$ is independent of the diffusion coefficient (Eq. 15). Table 3 clearly shows the importance of the $Da_{\textrm{c}}$ number on extraction efficiency, as the extraction efficiency doesn’t change much as long as the $Da_{\textrm{c}}$ remains constant.

**Fig. 4:**
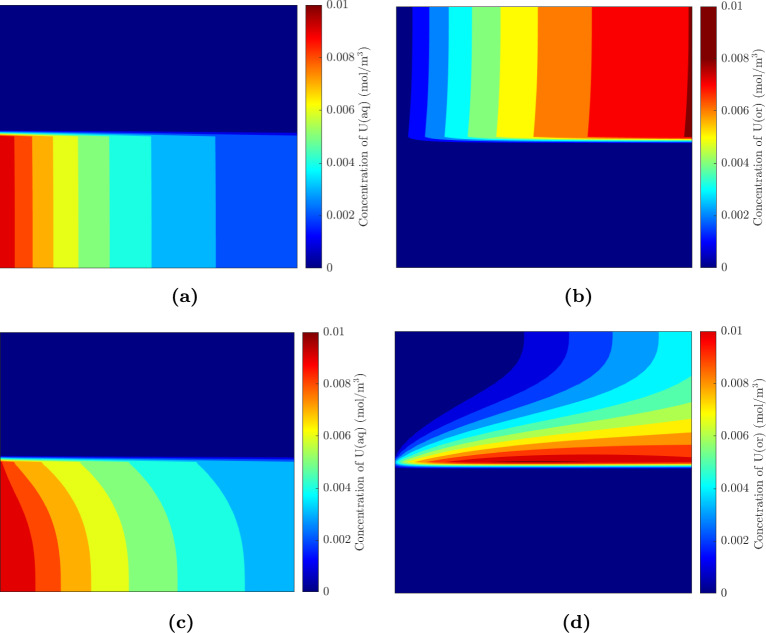
Simulated concentration profiles using the data from Hellé et al. (2015) but altering the diffusion coefficient such that **a**, **b** $D_{\textrm{aq}}$= 10$^{-7}$ $\hbox {m}^2$/s, $D_{\textrm{aq}}$= 10$^{-8}$ $\hbox {m}^2$/s and **c**, **d** $D_{\textrm{aq}}$= 10$^{-9}$ $\hbox {m}^2$/s, $D_{\textrm{aq}}$= 10$^{-10}$ $\hbox {m}^2$/s

The above results could be very useful in extraction studies for parallel flow when the diffusion coefficient is unknown. However, the obtained results do not mean that the diffusion coefficient has no influence altogether. The impact of the diffusion coefficient is more clearly observed in the concentration profiles. Figure 4 shows the concentration profiles of the fluids when $D_{\textrm{aq}}$= 10$^{-7}$ $\hbox {m}^2$/s, $D_{\textrm{aq}}$= 10$^{-8}$ $\hbox {m}^2$/s and $D_{\textrm{aq}}$= 10$^{-9}$ $\hbox {m}^2$/s, $D_{\textrm{aq}}$= 10$^{-10}$ $\hbox {m}^2$/s, and these can be compared with Fig. 2, where the actual experimental diffusion coefficients were used. When the diffusion coefficient is higher, the concentration gradients are less steep and more evenly dispersed. This can be observed in Fig. 4b, where the Uranium concentration near the walls is almost equal to that at the interface because the Uranium has quickly diffused along the width of the channel after the reaction had taken place at the interface. On the other hand, for lower diffusion coefficients, Uranium is concentrated near the interface in the organic fluid. In Fig. 4d, the Uranium concentration in the organic phase is much larger nearer to the interface than the channel wall, and this is clearly due to the low diffusion coefficient. The diffusion process is too slow to traverse the entire width of the channel, leaving Uranium to concentrate in the regions near the interface. Therefore, the diffusion coefficient influences the absolute distribution of Uranium, but has a low influence on the overall extraction efficiency in parallel flow-based mass transfer.

Table 3 showed that the diffusion coefficient has little impact on the extraction efficiency. To check if $Da_{\textrm{c}}$ has the maximum influence on extraction efficiency, we vary the channel width *H* in the next section.

#### Influence of the channel width

Numerous papers have studied the influence of the channel width on extraction efficiency for parallel flow (Farahani et al. 2021; Fries et al. 2008; Jovanović et al. 2012), and the overall conclusion of all these papers is that the extraction efficiency decreases with an increase in width. The larger interfacial area resulting from the larger width has been attributed as the main reason for this observation as this increases the diffusion time (The channel width is along the y direction as shown in Fig. 1). While this reason is indicative of what happens in channels with larger widths, it doesn’t provide the full picture as the dimensionless numbers are not included in the discussion. From Eqs. 12 and 13, we can see that both the Peclet and Biot number are influenced by the width, though Pe $\propto$ $\hbox {H}^2$ while Bi $\propto$ H. This means that if the average velocity remains constant in channels of different widths, the extraction efficiency should decrease with an increase in width, as the Pe number increases quicker than the Bi number.

**Table 4:** Influence of channel width on the extraction efficiency when average velocity is kept constant at $v_{\textrm{aq}}$=0.0138 m/s and $v_{\textrm{org}}$=0.011 m/s

**Width**($\mu$m)	$\mathbf {Pe_{\textrm{aq}}}$	$\mathbf {Pe_{\textrm{org}}}$	$\mathbf {Bi_{\textrm{aq}}}$	$\mathbf {Bi_{\textrm{org}}}$	$\mathbf {Da_{\textrm{c,aq}}}$	$\mathbf {Da_{\textrm{c,org}}}$	**EE(%)**
50	0.0202	0.195	0.08	0.8	3.950	4.100	84.38
100	0.0810	0.780	0.16	1.6	1.980	2.050	76.31
200	0.3240	3.120	0.32	3.2	0.990	1.025	56.41
300	0.7290	7.090	0.48	4.8	0.660	0.680	42.31
400	1.2960	12.48	0.64	6.4	0.495	0.512	33.72
500	2.0250	19.50	0.80	8.0	0.395	0.410	27.79
600	2.9160	28.08	0.96	9.6	0.333	0.343	23.65

Table 4 confirms our expectations of the extraction efficiency reducing with increasing width, as long as the average velocity remains the same. It also shows how the extraction efficiency changes with $Da_{\textrm{c}}$, as a higher $Da_{\textrm{c}}$ corresponds to a higher extraction efficiency. So we run simulations in channels of different widths with the flow rates of the fluids per unit depth (*uH*) being constant, thus changing the average velocity accordingly and keeping $Da_{\textrm{c}}$ constant. The reason for doing this is to confirm if the results observed in Table 3 are indeed due to the constant $Da_{\textrm{c}}$

Table 5 shows results similar to that of Table 3, where the extraction efficiency remains more or less constant as long as the $Da_{\textrm{c}}$ number remains constant. This shows the importance of the $Da_{\textrm{c}}$ number on the extraction efficiency, and why looking at merely the influence of the channel width doesn’t give the full picture unless we consider the influence of the dimensionless numbers. Considering the results from Tables 3, 4 and 5, we therefore look to develop a correlation for the extraction efficiency in terms of $Da_{\textrm{c}}$ in the next section as it is observed to have the maximal impact.

**Table 5:** Influence of channel width on the extraction efficiency when the flow rate is kept constant, and thus $Da_{\textrm{c}}$ is also constant

**Width** ($\mathbf {\mu }$m)	$\mathbf {Pe_{\textrm{aq}}}$	$\mathbf {Pe_{\textrm{org}}}$	$\mathbf {Bi_{\textrm{aq}}}$	$\mathbf {Bi_{\textrm{org}}}$	$\mathbf {Da_{\textrm{c,aq}}}$	$\mathbf {Da_{\textrm{c,org}}}$	**EE(%)**
50	0.0405	0.39	0.08	0.8	1.98	2.05	76.88
100	0.0810	0.78	0.16	1.6	1.98	2.05	76.31
200	0.1620	1.56	0.32	3.2	1.98	2.05	74.91
300	0.2430	2.34	0.48	4.8	1.98	2.05	73.27
400	0.3240	3.12	0.64	6.4	1.98	2.05	72.64
500	0.4050	3.90	0.80	8.0	1.98	2.05	71.56
600	0.4860	4.68	0.96	9.6	1.98	2.05	70.51

#### Extraction efficiency correlation

Here, we look to develop a correlation to understand how the extraction efficiency varies with the $Da_{\textrm{c}}$ number so that these results can be extended to other fluids in channels with rectangular cross-sections. Using dimensionless analysis, the extraction efficiency for interfacial mass transfer in a Y-Y microfluidic channel can be expressed as follows:

20 $$\begin{aligned} EE = f(Pe,Bi) = c_2 Pe^{\alpha }Bi^{\beta } \end{aligned}$$

where $c_2$ is a constant. Tables 3 and 5 showed that the extraction efficiency remains approximately the same even though the diffusion coefficient and, *uH* are changed. This implies that $\alpha = -\beta$, therefore Eq. 20 can be simplified to:

21 $$\begin{aligned} EE= c Da_{\textrm{c}}^{\beta } \end{aligned}$$

**Fig. 5:**
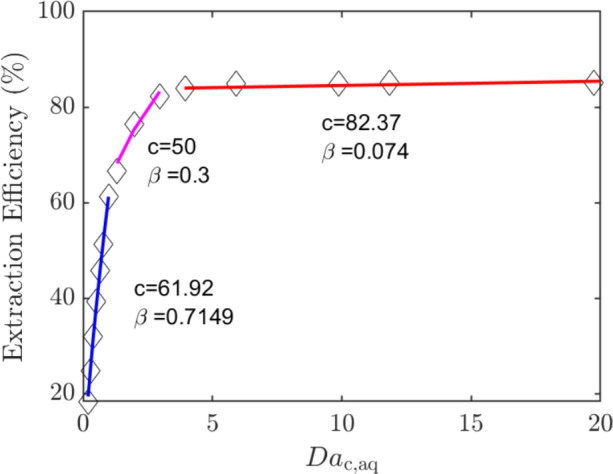
Influence of $Da_{\textrm{c}}$ on the extraction efficiency. The aqueous $Da_{\textrm{c}}$ is shown in the x-axis and the data is fitted using curves to observe the trend

We now plot the extraction efficiency against $Da_{\textrm{c}}$ to observe if it follows the above relation. From Fig. 5, we can see that Eq. 21 is followed for all the $Da_{\textrm{c}}$, but the values of *c* and $\beta$ are different for different regions. For $Da_{\textrm{c}} > 1$, the extraction efficiency increases significantly with the $Da_{\textrm{c}}$. The increase in extraction efficiency is reduced for $2<Da_{\textrm{c}}<5$, which appears to be a transition region, and then a saturation in the extraction efficiency is observed for $Da_{\textrm{c}}>5$. At low $Da_{\textrm{c}}$, not all the reactants have reacted as either the reaction rates are low, or they leave the chip early because of a high flow rate. As $Da_{\textrm{c}}$ increases, more reactants react quickly at the interface and therefore, more of the isotope is transferred to the organic phase. At a large $Da_{\textrm{c}}$, most of the reactants have already reacted, so much so that even a low flow rate has a minimal impact. It must be stressed that these results are only valid for a Y-Y channel of symmetric depth and rectangular cross-section, and for first-order reactions taking place at the interface only. It would be interesting to observe the trend for a channel of asymmetric depth such as the ones used in some papers (Smirnova et al. 2006; Ban et al. 2011), especially considering the utility of such channels in radioisotope transfer (Trapp et al. 2024).

To summarize, Tables 3, 4 and 5 show the influence of $Da_{\textrm{c}}$ on the extraction efficiency. A higher extraction efficiency is obtained at a higher $Da_{\textrm{c}}$, and if $Da_{\textrm{c}}$ does not change, the extraction efficiency is less affected regardless of whether the Pe or Bi number changes.

## Conclusion

Mass transfer with diffusion and a first-order reaction at the interface was studied for two fluids flowing parallel to each other. First, simulations were performed and validated using the data of Hellé et al. (2015). After validation, the influence of three dimensionless numbers—the Biot, Peclet and $Da_{\textrm{c}}$—was studied on the mass transfer. In general, a higher Biot number and a lower Peclet number are preferred. However, when both numbers are changed in such a way that the $Da_{\textrm{c}}$ is constant, the extraction efficiency remains approximately the same. A considerable increase in extraction efficiency is observed only when $Da_{\textrm{c}}$ increases. Finally, a relation for extraction efficiency was proposed in terms of $Da_{\textrm{c}}$ and extraction efficiency was plotted against $Da_{\textrm{c}}$. This relationship showed three different regions, where the extraction efficiency was observed to be low for low $Da_{\textrm{c}}$, higher as the $Da_{\textrm{c}}$ increases up to a value of 5, and saturated at $Da_{\textrm{c}}$ numbers higher than that.

The 2D model might involve simplifications, but this allows us to understand the role of each dimensionless number better, thereby paving the way for further studies in this vein. Clearly, there is scope for improvement, as the model looks only at 2D geometries and not 3D. As stated earlier, the main reason for this was to fix the position of the interface to avoid leakage. However, for channels with low aspect ratios, it would be better to use a 3D geometry to observe the role of these dimensionless numbers. The discussed results should also be extended to channels with different cross-sections such as circular microchannels (Ramji and Pushpavanam 2019) or channels with asymmetric depth (Trapp et al. 2024; Sudha et al. 2025) to characterize the role of geometry in mass transfer.

## Data Availability

Data will be made available on request.
